# Type 1 diabetes-related autoimmune antibodies in women with gestational diabetes mellitus and the long-term risk for glucose intolerance

**DOI:** 10.3389/fendo.2022.973820

**Published:** 2022-08-24

**Authors:** Kaat Beunen, Lies Vercauter, Paul Van Crombrugge, Carolien Moyson, Johan Verhaeghe, Sofie Vandeginste, Hilde Verlaenen, Chris Vercammen, Toon Maes, Els Dufraimont, Nele Roggen, Christophe De Block, Yves Jacquemyn, Farah Mekahli, Katrien De Clippel, Annick Van Den Bruel, Anne Loccufier, Annouschka Laenen, Roland Devlieger, Chantal Mathieu, Katrien Benhalima

**Affiliations:** ^1^ Department of Endocrinology, University Hospitals Gasthuisberg, KU Leuven, Leuven, Belgium; ^2^ Medicine, KU Leuven, Leuven, Belgium; ^3^ Department of Endocrinology, Onze Lieve Vrouw (OLV) Hospital Aalst-Asse-Ninove, Aalst, Belgium; ^4^ Department of Obstetrics and Gynecology, University Hospitals Gasthuisberg, KU Leuven, Leuven, Belgium; ^5^ Department of Obstetrics and Gynecology, OLV Hospital Aalst-Asse-Ninove, Aalst, Belgium; ^6^ Department of Endocrinology, Imelda Hospital, Bonheiden, Belgium; ^7^ Department of Obstetrics and Gynecology, Imelda Hospital, Bonheiden, Belgium; ^8^ Department of Endocrinology-Diabetology-Metabolism, Antwerp University Hospital, Edegem, Belgium; ^9^ Department of Obstetrics and Gynecology, Antwerp University Hospital, Edegem, Belgium; ^10^ Department of Endocrinology, Hospital St Jan Brussel, Brussel, Belgium; ^11^ Department of Obstetrics and Gynecology, Hospital St Jan Brussel, Brussel, Belgium; ^12^ Department of Endocrinology, General Hospital St Jan Brugge, Brugge, Belgium; ^13^ Department of Obstetrics and Gynecology, General Hospital St Jan Brugge, Brugge, Belgium; ^14^ Center of Biostatics and Statistical Bioinformatics, KU Leuven, Leuven, Belgium

**Keywords:** gestational diabetes mellitus, autoimmune antibodies, type 1 diabetes mellitus, pregnancy, follow-up, long-term risk, glucose intolerance

## Abstract

**Aims:**

To characterize women with gestational diabetes mellitus (GDM) positive for type 1 diabetes-related autoimmune antibodies (T1D-related autoantibodies) in pregnancy and to evaluate their risk for long-term glucose intolerance.

**Methods:**

In a multi-centric prospective cohort study with 1843 women receiving universal screening for GDM with a 75 g oral glucose tolerance test (OGTT), autoantibodies were measured in women with GDM: insulin autoantibodies (IAA), islet cell antibodies (ICA), insulinoma-associated protein-2 antibodies (IA-2A) and glutamic acid decarboxylase antibodies (GADA). Long-term follow-up ( ± 4.6 years after delivery) with a 75 g OGTT and re-measurement of autoantibodies was done in women with a history of GDM and autoantibody positivity in pregnancy.

**Results:**

Of all women with GDM (231), 80.5% (186) received autoantibody measurement at a mean of 26.2 weeks in pregnancy, of which 8.1% (15) had one positive antibody (seven with IAA, two with ICA, four with IA-2A and two with GADA). Characteristics in pregnancy were similar but compared to women without autoantibodies, women with autoantibodies had more often gestational hypertension [33.3% (5) vs. 1.7% (3), p<0.001] and more often neonatal hypoglycemia [40.0% (6) vs. 12.5% (19), p=0.012]. Among 14 of the 15 autoantibody positive women with an early postpartum OGTT, two had impaired fasting glucose (IFG). Of the 12 women with long-term follow-up data, four tested again positive for T1D-related autoantibodies (three positive for IA-2A and one positive for ICA and IAA). Five women were glucose intolerant at the long-term follow-up of which two had IA-2A (one had IFG and one had T1D) and three without autoantibodies. There were no significant differences in long-term characteristics between women with and without autoantibodies postpartum.

**Conclusions:**

Systematic screening for T1D-related autoantibodies in GDM does not seem warranted since the low positivity rate for autoantibodies in pregnancy and postpartum. At 4.6 years postpartum, five out of 12 women were glucose intolerant but only two still had autoantibodies. In women with clinically significant increased autoantibody levels during pregnancy, postpartum autoantibody re-measurement seems useful since the high risk for further increase of autoantibody levels.

## Introduction

Gestational diabetes mellitus (GDM) is a common medical condition during pregnancy. It is defined as glucose intolerance diagnosed in the second or third trimester that was not clearly overt diabetes in early pregnancy (1). GDM raises the risk of pregnancy complications such as gestational hypertension, preeclampsia, preterm delivery, and large for gestational age (LGA) infants (2–5). Pregnancy outcomes can be improved by GDM screening and treatment between 24-28 weeks of pregnancy (4, 5). A universal one-step screening approach with 2-h 75 g oral glucose tolerance test (OGTT) between 24-28 weeks and using stringent diagnostic criteria is currently recommended by the ‘International Association of Diabetes and Pregnancy Study Groups’ (IADPSG) to diagnose GDM (3, 6). Generally, glucose levels are restored to normal shortly after delivery. However, women with a history of GDM are at increased risk of developing future type 2 diabetes (T2D), cardiovascular disorders, and metabolic syndrome compared to normal glucose tolerant (NGT) women (7–10).

Not all gestational hyperglycemia has the same etiology. Gestational hyperglycemia develops when the β-cell insulin response, normally adapting to increased physiological needs and functional demands of pregnancy, is inadequate (11). GDM screening strategies mainly focus on evaluating glucose homeostasis based on diagnostic criteria rather than reflecting the underlying pathophysiology. However, the underlying pathophysiology might contribute to adverse pregnancy outcomes (12).

Sometimes, GDM masquerades undetected autoimmune type 1 diabetes mellitus (T1D) (13). In a small percentage of women with GDM, usually <10%, GDM diagnosis is associated with autoimmunity against pancreatic β-cells (i.e. autoimmune destruction of β-cells), following expression of T1D-related autoimmune antibodies (autoantibodies) such as insulin autoantibodies (IAA), islet cell antibodies (ICA), insulinoma-associated protein-2 antibodies (IA-2A), glutamic acid decarboxylase antibodies (GADA), and zinc transporter 8 antibodies (ZnT8A) (13–15). Data on the exact prevalence and levels of individual autoantibodies in GDM women remain inconclusive. Some studies showed no differences in pregnancy outcomes between GDM women with and without autoantibodies (16–18). This may imply that maternal hyperglycemia, regardless of the cause, is the main determinant of adverse pregnancy outcomes (13). Nevertheless, women with a history of GDM and autoantibody positivity in pregnancy have a higher risk to develop future impaired glucose regulation, T1D or Latent Autoimmune Diabetes of Adulthood (LADA) (13, 15, 19–22). Identification of T1D-related autoantibodies in GDM women might therefore facilitate better understanding of the pathophysiology underlying gestational hyperglycemia and contribute to more accurate classification of GDM (15, 16). Moreover, identification of these women might optimize antenatal management strategies to avoid adverse pregnancy outcomes related to T1D, or acute onset of diabetes with diabetic ketoacidosis (15, 16). So far, there are no clear recommendations in which women with GDM it would be clinically relevant to screen for autoantibodies. In addition, data on the long-term prevalence of autoantibodies after GDM and the risk to develop glucose intolerance postpartum are limited (13, 14). We aimed therefore to characterize women with GDM and T1D-related autoantibodies in pregnancy and to evaluate their long-term risk for glucose intolerance.

## Patients and methods

### Study design and setting

This is a sub-analysis of the ‘Belgian Diabetes in Pregnancy’ (BEDIP-N) study, a multi-centric prospective cohort study previously described in detail (23–26). The study was registered in ClinicalTrials.gov (NCT02036619). The study protocol received approval by Institutional Review Boards of all participating centers and all investigations have been carried out in accordance with the principles of the Declaration of Helsinki as revised in 2008. Between April 2014 and March 2017, women between 18–45 years who presented for prenatal care between 6–14 weeks of pregnancy in two university and four non-university hospitals in Belgium, were invited to participate in the study. Before inclusion, participants provided informed consent. In the first trimester, women were screened for overt diabetes and impaired fasting glycemia (IFG) in early pregnancy, as defined by the American Diabetes Association (ADA), using fasting plasma glucose (FPG) (27). Participants with FPG <100 mg/dL were universally screened for GDM between 24-28 weeks, using both a non-fasting 50 g glucose-challenge test (GCT) and 2-h 75 g OGTT. Participants and health care providers were blinded for the GCT result, so all women received an OGTT irrespective of the GCT result (24, 25). GDM diagnosis was based on IADPSG criteria. Women with GDM were treated according to ADA recommended glycemic targets (27, 28). If targets were not reached within two weeks after start of lifestyle measures, insulin treatment was started. GDM women were invited 6–16 weeks postpartum to receive a 2-h 75 g OGTT. Glucose intolerance postpartum [diabetes, IFG and/or impaired glucose tolerance (IGT)] was defined using ADA criteria (24, 27).

### Study visits and assessments

At the first antenatal visit (6-14 weeks), baseline characteristics and obstetrical history were collected (24). Minority ethnic background was defined as having at least one parent from non-Caucasian origin. At first visit and at the OGTT, anthropometric measurements were collected and self-administered questionnaires were completed (24). Blood pressure (BP) was measured twice at 5 min intervals with an automatic BP monitor (24). Hypertension was defined as systolic BP (SBP) ≥140 mmHg and/or diastolic BP (DBP) ≥90 mmHg. Overweight was defined as BMI ≥25 kg/m^2^ and obesity as BMI ≥30 kg/m^2^ based on body mass index (BMI) at first visit. FPG, insulin, lipid profile [total cholesterol, high-density (HDL), and low-density lipoprotein (LDL) cholesterol and triglycerides (TG)], and hemoglobin A1c (HbA1c) were measured fasting between 6-14 weeks (24). At the GCT, non-fasting glycemia was evaluated, followed by consumption of a 50 g glucose load to evaluate 1-h plasma glucose. At the OGTT, fasting lipid profile and HbA1c were determined. Glucose and insulin levels were measured fasting, at 30, 60, and 120 min (24). Analyses of FPG at first visit and glucose measurements of the OGTT were performed locally at each center, while analyses of GCT samples, insulin, lipids, and HbA1c were performed centrally at the UZ Leuven laboratory. Extra serum samples were collected in GDM women to detect autoantibodies according to routine guidelines of the ‘Belgian Diabetes Registry’ (BDR; new diagnosis of diabetes or GDM in women <40 years). Autoantibodies were analyzed by the laboratory of UZ Brussel using liquid-phase radiobinding assay for IAA, IA-2A, and GADA detection and indirect immunofluorescence for ICA detection, as described previously (29–33). Cut-off values for antibody-positivity were determined as the 99th percentile of antibody levels in 761 non-diabetic controls, after removing outliers. The upper normal limit was <0.6% binding for IAA, <12 Juvenile Diabetes Foundation units (JDF U) for ICA, <1.4 WHO U/mL for IA-2A, and <23 WHO U/mL for GADA. Internal quality controls (negative, positive low and/or high) are applied on the detection methods for all autoantibodies at least once per run. Once per two year, an external quality control named Islet Autoantibody Standardization Program (IASP) is applied on the detection method for GADA and IA-2A. At the early postpartum OGTT, anthropometric measurements were performed and self-administered questionnaires were completed (24). Continuation of breastfeeding during the OGTT was recorded. Glucose and insulin levels from the OGTTs were used to calculate different indices of insulin sensitivity [Matsuda index and Homeostatic Model Assessment for Insulin Resistance (HOMA-IR)] and β-cell function [Homeostatic Model Assessment for β-cell function (HOMA-B), insulinogenic index divided by HOMA-IR and insulin secretion-sensitivity index-2 (ISSI-2)] (24, 34–38). These measures have been validated for use in women with GDM and have been used in the BEDIP-N study (24, 34–38).

### Pregnancy and delivery outcomes

We collected pregnancy outcome data such as gestational age, preeclampsia (*de novo* BP ≥140/90 mmHg >20 weeks with proteinuria or signs of end-organ dysfunction), gestational hypertension (*de novo* BP ≥140/90 mmHg >20 weeks), type of labor and delivery, birth weight, macrosomia (>4 kg), birth weight ≥4.5 kg, LGA and small for gestational age (SGA) defined as birth weight >P90 and <P10 according to standardized Flemish birth charts adjusted for the baby’s sex and parity, respectively (39), preterm delivery (<37 weeks), neonatal hypoglycemia (<40 mg/dL), and neonatal intensive care unit (NICU) admission (24). NICU admission was decided in line with normal routine care by the local neonatologist. Gestational weight gain in early pregnancy was calculated as the difference in weight between the first antenatal visit and the OGTT, and total gestational weight gain as the difference in weight between the first antenatal visit and delivery. Excessive total gestational weight gain was defined according to 2009 Institute of Medicine guidelines (40).

### Long-term follow-up of women with a history of GDM and autoantibodies in pregnancy

Long-term follow-up in women with GDM and autoantibodies in pregnancy was standardized across all centers by performing a 2-h 75 g OGTT and re-measurement of autoantibodies. The following data were collected: results of the glucose and insulin levels at the OGTT, HbA1c, fasting C-peptide, lipid profile (total cholesterol, HDL, LDL, and TG), levels of autoantibodies measured by the BDR [IAA, ICA, IA-2A, and GADA detection as described above, and ZnT8A detection using liquid-phase radiobinding assay with upper normal limit <1.02% binding and internal and external (IASP) quality control], weight, BMI, waist circumference, BP, different indices of insulin sensitivity [Matsuda index, HOMA-IR, and reciprocal HOMA-IR (1/HOMA-IR)], and β-cell function (HOMA-B, the insulinogenic index/HOMA-IR, ISSI-2, and Stumvoll index) (24, 34–38, 41, 42).

### Statistical analysis

Descriptive statistics were presented as frequencies and percentages for categorical variables and means with standard deviations or medians with interquartile range for continuous variables. Categorical variables were analyzed using the Chi-square test or Fisher exact test in case of low (<5) cell frequencies, whereas continuous variables were analyzed using the Kruskal-Wallis test for not normally distributed variables or One-way ANOVA test for normally distributed variables. A p-value <0.05 was considered significant. Analyzes were performed by statistician A. Laenen using SAS version 9.4.

## Results

### Prevalence of T1D-related autoantibodies in GDM women during pregnancy and postpartum

**Figure 1:**
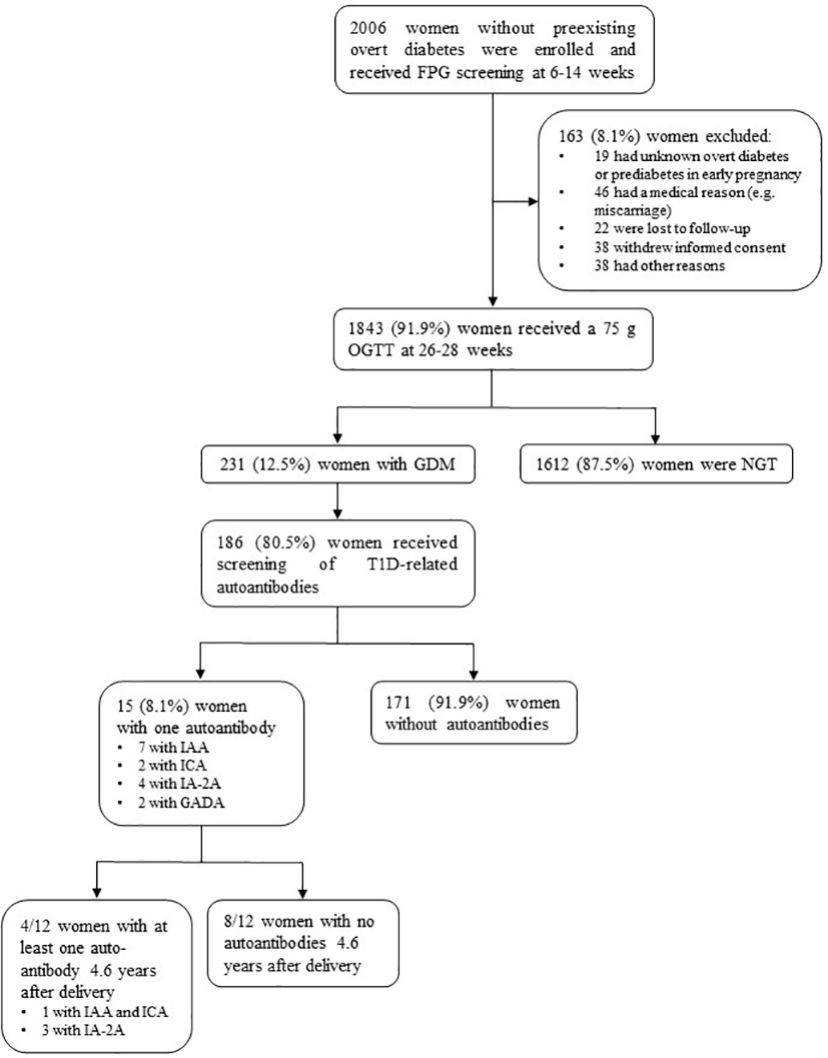
Flow diagram of the BEDIP-N study cohort. FPG, fasting plasma glucose; OGTT, oral glucose tolerance test; GDM, gestational diabetes mellitus; NGT, normal glucose tolerant; T1D, type 1 diabetes mellitus; IAA, insulin autoantibodies; ICA, islet cell antibodies; IA-2A, insulinoma-associated protein-2 antibodies; GADA, glutamic acid decarboxylase antibodies.

In total, 1843 women received GDM screening between 26-28 weeks using an OGTT (**Figure 1**). Of all women with GDM (231), 80.5% (186) were screened for T1D-related autoantibodies at a mean gestational age of 26.2 weeks, of which 8.1% (15) had one positive autoantibody [seven with IAA, two with ICA, four with IA-2A, and two with GADA] (**Table 1**). Of these 15 women, ten had borderline increased autoantibodies (at or just above the upper limit of normal). Of the 12 women with long-term follow-up data ±4.6 years after delivery, four tested again positive for autoantibodies: three were positive for IA-2A and one was positive for both ICA and IAA (**Table 1**). Of all women with borderline increased autoantibodies in pregnancy and follow-up data, none had clinically significant increased autoantibodies at long-term follow-up (**Table 1**). In contrast, of the five women with clinically significant increased autoantibodies in pregnancy [two with IAA, two with IA-2A, and one with GADA], four had higher autoantibodies levels at long-term of which two developed glucose intolerance (one with IFG and one with T1D).

**Table 1:** Levels of T1D-related autoantibodies in women with GDM in pregnancy and at long-term follow-up.

	1	2	3	4	5	6	7	8	9	10	11	12	13	14	15
**During pregnancy**															
ICA (≤11.9 JDF U)	**12**	Neg	Neg	Neg	Neg	Neg	Neg	Neg	**12**	Neg	Neg	Neg	Neg	Neg	Neg
IAA (<0.6% binding)	0.4	0.2	**3.3**	0.5	0.3	**2.0**	0.1	0.4	0.3	**0.7**	**0.6**	**0.8**	0.2	**0.7**	**0.8**
GADA (<23 U/mL)	2.2	1.5	2.3	6.4	4.7	2.0	**79.5**	7.9	0.1	0.6	17	0.1	**26.1**	1.6	0.1
IA-2A (<1.4 U/mL)	0.2	**2.9**	0.1	**1.4**	**3.0**	0.1	0.7	**1.5**	0.1	0.1	0.1	0.1	0.3	0.1	0.1
**At long-term follow-up**															
Follow-up after delivery (years)	6	6	2	6	5	5	4	3	3	4	6	5	/	/	/
ICA (≤11.9 JDF U)	**12**	Neg	**200**	Neg	Neg	Neg	Neg	Neg	**12**	Neg	Neg	Neg	/	/	/
IAA (<0.6% binding)	0.5	0.4	**2.7**	0.3	0.4	0.2	0.1	0.2	0.4	0.2	0.3	0.5	/	/	/
GADA (<23 U/mL)	<0.1	0.1	1	7.7	3.7	7.3	0.1	0.6	<0.1	0.6	2.6	<1.0	/	/	/
IA-2A (<1.4 U/mL)	<0.1	**15**	0.1	0.3	**4.4**	**3.1**	0.1	0.1	<0.1	<0.1	0.2	/	/	/	/
ZnT8A (<1.02% binding)	/	0.2	0.6	0.5	0.2	0.3	0.3	0.2	/	/	0.5	/	/	/	/

ICA, islet cell antibodies; JDF, U Juvenile Diabetes Foundation units; IAA, insulin autoantibodies; GADA, glutamic acid decarboxylase antibodies; IA-2A, insulinoma-associated protein-2 antibodies; ZnT8A, zinc transporter 8 antibodies; Neg negative result. Numbers 1-12 represent the GDM women with autoantibodies that also had a long-term follow-up visit, numbers 13-15 represent GDM women with autoantibodies that had no long-term follow up visit. (Borderline) positive T1D-related autoantibodies are indicated bold. ‘/’ means that not enough sample was available to measure the T1D-related autoantibody or that samples were not collected.

### Characteristics in pregnancy, early and long-term postpartum

At baseline and at 26-28 weeks in pregnancy, characteristics were similar between GDM women with autoantibodies (15) and GDM women without autoantibodies (171) (**Table 2**, and **Appendix 1**). Compared to GDM without autoantibodies, GDM with autoantibodies had more often gestational hypertension [33.3% (5) vs. 1.7% (3), p<0.001] and more often neonatal hypoglycemia [40.0% (6) vs. 12.5% (19), p=0.012] (**Table 2**). The rate of glucose intolerance at ±12.9 weeks postpartum (early postpartum) was not significantly different between GDM women with and without autoantibodies: 14.3% (2) of GDM women with autoantibodies (one with IA-2A and one with IAA) were diagnosed with IFG, while 18.3% (28) of all GDM women without autoantibodies had glucose intolerance (**Table 3**). Besides a lower fasting LDL-cholesterol in GDM women with autoantibodies, there were no significant differences in early postpartum characteristics between both groups (**Table 3**, **Appendix 2, 3**). Long-term follow-up data were available for 12 of the 15 women with a history of GDM and autoantibodies in pregnancy. One woman did not attend both the early postpartum and long-term follow-up OGTT, and two women who were NGT at early postpartum did not attend the long-term follow-up OGTT. At long-term follow-up, five women were glucose intolerant of which two with IA-2A (one had IFG and one had T1D) and three without autoantibodies (one had IGT, one had both IFG and IGT, and one had T2D) (**Table 1** and **Appendix 3**). Indices of insulin resistance, beta-cell function and fasting C-peptide were similar between women with and without autoantibodies at long-term (**Table 4**). Other long-term characteristics were also not significantly different between both groups (**Table 4** and **Appendix 3**).

**Table 2:** Comparison of characteristics and pregnancy outcomes between GDM with autoantibodies (group 1), GDM without autoantibodies (group 2) and NGT women (group 3).

	GDM with autoantibodiesN=15 (0.8%)	GDM without autoantibodies N=171 (9.5%)	NGTN=1612 (89.7%)	p-value		
				1 vs 2	1 vs 3	2 vs 3
**General**						
Age (years)	32.9 ± 4.8	31.7 ± 4.4	30.6 ± 3.9	0.298	**0.024**	**<0.001**
% Minority ethnic background	13.3 (2)	16.5 (28)	8.2 (132)	1.000	0.357	**0.001**
% Smoking before pregnancy	26.7 (4)	40.8 (69)	28.5 (457)	0.410	1.000	**0.001**
% Smoking during pregnancy	0.0 (0)	6.5 (11)	3.2 (52)	0.604	1.000	**0.045**
% First degree family history of diabetes	6.7 (1)	19.8 (33)	11.8 (185)	0.310	1.000	**0.005**
% First degree family history of GDM	0.0 (0)	8.2 (13)	4.0 (60)	0.602	1.000	**0.022**
% History of GDM ^c^	16.7 (1)	30.0 (27)	5.3 (40)	0.668	0.282	**<0.001**
% History of IGT ^a^	0.0 (0)	3.3 (5)	1.1 (15)	1.000	1.000	**0.043**
**6-14 weeks visit**						
BMI (kg/m²)	27.0 ± 5.4	26.7 ± 5.4	24.4 ± 4.5	0.868	**0.027**	**<0.001**
% Overweight % Obesity	26.7 (4) 26.7 (4)	31.2 (53) 24.1 (41)	24.8 (398) 11.0 (177)	0.970	0.114	**<0.001**
Waist circumference (cm)	90.1 ± 12.4	91.5 ± 12.8	86.5 ± 10.9	0.683	0.204	**<0.001**
% Waist ≥80 cm	93.3 (14)	81.5 (132)	74.1 (1144)	0.156	0.196	**0.002**
Systolic blood pressure (mmHg)	117.4 ± 9.1	116.8 ± 11.8	114.8 ± 10.4	0.841	0.335	**0.020**
Diastolic blood pressure (mmHg)	73.5 ± 7.0	72.1 ± 8.7	70.3 ± 8.1	0.558	0.134	**0.001**
Fasting glycemia (mg/dL)	86.0 (84.0 – 90.0)	85 (80.0 – 89.0)	81 (78.0 – 85.0)	0.242	**<0.001**	**<0.001**
HOMA-IR	10.0 (9.5 – 19.9)	10.9 (7.9 – 16.8)	9.1 (6.5 – 12.9)	0.689	**0.040**	**<0.001**
HbA1c (mmol/mol and %)	32.0 (30.0 – 36.0) 5.1 (4.9 – 5.4)	32.0 (30.0 – 34.0) 5.1 (4.9 – 5.3)	31.0 (29.0 – 32.0) 5.0 (4.8 – 5.1)	0.495	**0.044**	**<0.001**
Fasting total cholesterol (mg/dL)	177.0 (157.0 – 205.0)	186.0 (164.0 – 211.0)	180.0 (161.0 – 203.0)	0.252	0.606	**0.017**
Fasting TG (mg/dL)	104.0 (70.0 –118.0)	98.0 (81.0 – 133.0)	88.0 (71.0 – 111.0)	0.603	0.280	**<0.001**
**24-28 weeks visit**						
BMI (kg/m²)	29.0 ± 5.1	29.3 ± 5.3	26.9 ± 4.4	0.854	0.084	**<0.001**
% Overweight % Obesity	42.9 (6) 28.6 (4)	42.1 (69) 37.2 (61)	40.1 (629) 21.1 (332)	0.855	0.677	**<0.001**
Systolic blood pressure (mmHg)	116.7 ± 10.3	115.4 ± 11.7	113.1 ± 10.1	0.670	0.167	**0.006**
Diastolic blood pressure (mmHg)	72.0 ± 8.1	69.1 ± 8.1	67.0 ± 7.9	0.189	**0.015**	**<0.001**
Glucose non-fasting 0 min on GCT (mg/dL)	105.0 ± 27.4	98.0 ± 20.5	88.0 ± 15.8	0.218	**0.004**	**<0.001**
Glucose 60 min on GCT (mg/dL)	145.9 ± 21.5	143.4 ± 29.7	116.9 ± 25.5	0.754	**<0.001**	**<0.001**
Fasting glycemia (mg/dL)	91.0 (84.0 – 94.0)	85.0 (78.0 – 92.0)	78.0 (74.0 – 82.0)	0.081	**<0.001**	**<0.001**
30 min glucose OGTT (mg/dL)	160.0 (130.0 – 177.0)	149.0 (134.0 – 163.0)	124.0 (112.0 – 137.0)	0.381	**<0.001**	**<0.001**
1-hour glucose OGTT (mg/dL)	178.0 (157.0 – 192.0)	172.5 (154.0 – 186.0)	123.0 (107.0 – 141.0)	0.241	**<0.001**	**<0.001**
2-hour glucose OGTT (mg/dL)	151.0 (142.0 – 169.0)	156.0 (134.0 – 167.0)	108.0 (92.0 – 124.0)	0.850	**<0.001**	**<0.001**
HbA1c (mmol/mol and %)	32.0 (30.0 – 34.0) 5.1 (4.9 – 5.3)	32.0 (30.0 – 34.0) 5.1 (4.9 – 5.3)	30.0 (29.0 – 32.0) 4.9 (4.8 – 5.1)	0.786	**0.005**	**<0.001**
Matsuda insulin sensitivity	0.3 (0.3 – 0.4)	0.4 (0.2 – 0.5)	0.6 (0.4 – 0.8)	0.510	**<0.001**	**<0.001**
HOMA-IR	17.0 (13.4 – 21.4)	17.3 (11.3 – 28.5)	11.9 (8.6 – 16.8)	0.986	**0.003**	**<0.001**
HOMA-B	1184.0 (814.1 – 1617.4)	1433.8 (1046.4 – 2065.1)	1591.3 (1133.9 – 2286.7)	0.115	**0.016**	**0.013**
ISSI-2	0.08 (0.05 – 0.11)	0.09 (0.04 – 0.15)	0.14 (0.08 – 0.25)	0.587	**0.003**	**<0.001**
Insulinogenic index/HOMA-IR	0.2 (0.1 – 0.3)	0.2 (0.2 – 0.3)	0.3 (0.2 – 0.5)	0.682	**<0.001**	**<0.001**
Fasting TG (mg/dL)	179.0 (145.0 – 203.0)	184.0 (147.0 – 233.0)	160.0 (128.0 – 202.0)	0.513	0.241	**<0.001**
**Delivery**						
Total weight gain (first visit-delivery) (kg)	8.3 ± 4.6	8.6 ± 5.0	12.2 ± 5.0	0.803	**0.003**	**<0.001**
% Excessive weight gain	26.7 (4)	18.4 (28)	30.9 (437)	0.573	0.142	**<0.001**
Gestational age (weeks)	38.4 ± 1.6	38.9 ± 1.5	39.3 ± 1.6	0.281	**0.040**	**0.001**
% Gestational hypertension	33.3 (5)	1.7 (3)	4.2 (68)	**<0.001**	**<0.001**	0.148
% Preterm delivery	20.0 (3)	6.4 (11)	5.4 (86)	0.090	**0.045**	0.594
% Induction labor	46.7 (7)	36.3 (62)	25.9 (416)	0.420	0.079	**0.005**
% CS (total)	26.7 (4)	29.2 (50)	20.2 (324)	1.000	0.522	**0.008**
% Neonatal hypoglycemia <40 mg/dL ^b^	40.0 (6)	12.5 (19)	4.0 (41)	**0.012**	**<0.001**	**<0.001**
% NICU admission	20.0 (3)	15.2 (26)	9.6 (153)	0.708	0.171	**0.032**

GDM, gestational diabetes mellitus; NGT, normal glucose tolerance; IGT, impaired glucose tolerance; BMI, Body Mass Index; HOMA-IR, Homeostatic Model Assessment for Insulin Resistance; HbA1c, glycated hemoglobin; TG, triglycerides; GCT, glucose challenge test; OGTT, oral glucose tolerance test; HOMA-B, Homeostatic Model Assessment for β-cell function; ISSI-2, insulin secretion-sensitivity index-2; CS, caesarean section; NICU, neonatal intensive care unit. Overweight BMI ≥25-29.9 kg/m^2^; Obesity BMI ≥30 kg/m^2^. Categorical variables are presented as frequencies % (n); continuous variables are presented as mean ± SD if normally distributed and as median ± IQR if not normally distributed; differences are considered significant at p-value <0.05 and are indicated in bold.

a For these variables, data were missing in 10–15% of all participants.

b For these variables, data were missing in 25–35% of all participants.

c For these variables, data were missing in 50–55% of all participants.

**Table 3:** Comparison of characteristics between GDM with autoantibodies and GDM without autoantibodies at the early postpartum OGTT.

	GDM with autoantibodies N=14 (93.3%)	GDM without autoantibodies N=153 (89.5%)	p-value
% Present at OGTT	93.3 (14)	89.5 (153)	1.000
Time after delivery (weeks)	12.9 ± 1.6	14.6 ± 4.2	0.154
BMI (kg/m²)	25.5 (23.8 – 31.4)	26.0 (22.7 – 29.3)	0.566
% Overweight % Obesity	64.3 (9) 35.7 (5)	57.0 (85) 20.8 (31)	0.779 0.195
Waist circumference (cm)	89.0 (83.0 – 106.0)	91.0 (85.0 – 99.0)	0.488
% Waist ≥80 cm	92.3 (12)	83.7 (118)	0.459
Systolic blood pressure (mmHg)	115.7 (111.5 – 119.0)	116.0 (109.0 – 125.0)	0.541
Diastolic blood pressure (mmHg)	72.7 (68.0 – 76.0)	72.5 (67.0 – 78.0)	0.777
Fasting glycemia (mg/dL)	87.0 (86.0 – 92.0)	87.0 (83.0 – 93.0)	0.456
30 min glucose OGTT (mg/dL)	137.5 (123.0 – 156.0)	141.0 (124.0 – 156.0)	0.998
1-hour glucose OGTT (mg/dL)	140.0 (114.0 – 157.0)	128.0 (106.0 – 153.0)	0.472
2-hour glucose OGTT (mg/dL)	99.0 (88.0 – 109.0)	100.0 (90.0 – 120.0)	0.293
% Glucose intolerance IFG IGT IFG+IGT	14.3 (2) 0.0 (0) 0.0 (0)	5.9 (9) 11.1 (17) 1.3 (2)	0.371
HbA1c (mmol/mol and %)	33.0 (31.0 – 37.0) 5.2 (5.0 – 5.5)	33.0 (32.0 – 37.0) 5.2 (5.1 – 5.5)	0.625
Matsuda insulin sensitivity	0.6 (0.4 – 0.8)	0.7 (0.4 –1.0)	0.720
HOMA-IR	10.3 (8.4 – 22.0)	11.2 (7.8 – 17.9)	0.861
HOMA-B	675.0 (582.4 – 1317.9)	784.5 (554.7 – 1163.1)	0.950
ISSI-2	0.19 (0.07 – 0.35)	0.22 (0.10 – 0.45)	0.551
Insulinogenic index/HOMA-IR	2.4 (2.0 – 3.7)	2.6 (2.0 – 3.6)	0.778
Stumvoll index	142.6 (-164.6 – 807.5)	44.3 (-225.3 – 382.9)	0.465
Fasting total cholesterol (mg/dL)	172.5 (134.0 – 196.0)	182.0 (164.0 – 203.0)	0.153
Fasting HDL (mg/dL)	55.5 (52.0 – 69.0)	57.0 (48.0 – 69.0)	0.838
Fasting LDL (mg/dL)	77.0 (66.0 – 121.0)	104.0 (86.0 – 124.0)	**0.028**
Fasting TG (mg/dL)	69.5 (56.0 – 82.0)	79.0 (60.0 – 108.0)	0.194
% Breastfeeding	78.6 (11)	83.9 (125)	0.705

GDM, gestational diabetes mellitus; OGTT, oral glucose tolerance test; BMI, Body Mass Index; IFG, impaired fasting glycemia; IGT, impaired glucose tolerance; HbA1c, glycated hemoglobin; HOMA-IR, Homeostatic Model Assessment for Insulin Resistance; HOMA-B, Homeostatic Model Assessment for β-cell function; ISSI-2, insulin secretion-sensitivity index-2; HDL, high-density lipoprotein; LDL, low-density lipoprotein; TG, triglycerides. Overweight BMI ≥25 kg/m^2^; Obesity BMI ≥30 kg/m^2^. Categorical variables are presented as frequencies % (n); continuous variables are presented as mean ± SD if normally distributed and as median ± IQR if not normally distributed; differences are considered significant at p-value <0.05 and are indicated in bold.

**Table 4:** Comparison of long-term follow-up data among women with a history of GDM and T1D-related autoantibodies in pregnancy (n=12).

	Late autoantibody positive GDM women N=4 (33.3%)	Late autoantibody negative GDM women N=8 (66.7%)	p-value
Time after delivery (years)	5.0 (3.5 – 5.5)	4.5 (3.5 – 6.0)	1.000
BMI (kg/m²)	26.7 (24.4 – 27.7)	30.6 (28.1 – 37.6)	0.126
% Overweight % Obesity	66.7 (2) 0.0 (0)	87.5 (7) 50.0 (4)	0.491 0.236
Waist circumference (cm) ^b^	80.5 (78.8 – 82.5)	106.8 (83.7 – 107.0)	0.177
% Waist ≥80 cm ^b^	66.7 (2/3)	80.0 (4/5)	1.000
Systolic blood pressure (mmHg) ^b^	137.0 (110.0 – 138.0)	138.5 (130.0 – 147.0)	0.519
Diastolic blood pressure (mmHg) ^b^	85.0 (74.0 – 88.0)	86.0 (78.0 – 88.0)	0.696
Fasting glycemia (mg/dL)	95.0 (86.5 – 99.5)	94.5 (91.5 – 98.0)	0.865
30 min glucose OGTT (mg/dL)	146.0 (117.5 – 171.5)	148.0 (130.0 – 164.5)	0.932
1-hour glucose OGTT (mg/dL)	149.0 (140.5 – 182.5)	163.0 (138.0 – 186.0)	0.671
2-hour glucose OGTT (mg/dL)	105.0 (99.0 – 154.5)	138.5 (115.0 – 173.5)	0.203
% Glucose intolerance IFG IGT IFG+IGT	25.0 (1/4) 25.0 (1/4) 0.0 (0/4)	0.0 (0/8) 12.5 (1/8) 25.0 (2/8)	0.547
HbA1c (mmol/mol and %) ^a^	36.5 (36.0 – 37.5) 5.4 (5.4 – 5.5)	36.0 (33.0 – 40.0) 5.4 (5.2 – 5.8)	0.830 1.000
Fasting C-peptide (nmol/L) ^b^	2.2 (1.0 – 3.0)	1.9 (0.8 – 2.3)	1.000
Matsuda insulin sensitivity ^b^	0.3 (0.3 – 0.5)	0.3 (0.2 – 0.6)	0.665
HOMA-IR ^b^	18.7 (12.2 – 24.6)	23.0 (14.0 – 45.3)	0.470
HOMA-B ^b^	974.8 (786.3 – 1120.1)	1454.5 (953.6 – 1650.9)	0.312
ISSI-2 ^b^	0.06 (0.04 – 0.13)	0.06 (0.5 – 0.16)	0.470
Insulinogenic index/HOMA-IR ^b^	0.2 (0.1 – 0.2)	0.3 (0.1 – 0.5)	0.885
Stumvoll index ^b^	710.4 (334.9 – 1029.5)	957.8 (351.6 – 1169.4)	0.885

GDM, gestational diabetes mellitus; BMI, Body Mass Index; OGTT, oral glucose tolerance test; IFG, impaired fasting glycemia; IGT, impaired glucose tolerance; HbA1c, glycated hemoglobin; HOMA-IR, Homeostatic Model Assessment for Insulin Resistance; HOMA-B, Homeostatic Model Assessment for β-cell function; ISSI-2, insulin secretion-sensitivity index-2. Overweight BMI ≥25 kg/m^2^; Obesity BMI ≥ 30 kg/m^2^. Categorical variables are presented as frequencies % (n); continuous variables are presented as median ± IQR; differences are considered significant at p-value <0.05 and are indicated in bold.

a For these variables, data were missing in 10–20% of all participants.

b For these variables, data were missing in 25–35% of all participants.

## Discussion

Controversy still exists regarding the clinical relevance of screening for T1D-related autoantibodies during GDM pregnancy. In this large Belgian cohort, overall prevalence of autoantibodies in GDM women was 8.1%, confirming previous findings. Surprisingly, IAA was the most frequent positive autoantibody (3.8%), which is in contrast with previous observations, rarely reporting IAA in this population (14, 43). GADA positivity (1.1%) and IA-2A frequency (2.1%) in our cohort was in accordance with previous reports (0-10.8% and 0-6.2%, respectively) (13, 44). The rate of ICA positivity was rather low (1.1%). However, ICA frequency varies considerably between studies (1-35%) due to use of a not-standardized assay and consequently yielding numerous false positive results (13, 15, 16). Variations in autoantibody positivity rate in GDM women can be explained by the type of autoantibodies measured, the GDM diagnostic criteria, the ethnicity, and the T1D risk of the background population.

Antibody levels are generally lower in GDM compared to newly diagnosed T1D cases (13–15). Gestational immunomodulation may alter presence and levels of autoantibodies, possibly resulting in false negative results in pregnancy (13, 15, 21). Consequently, postpartum autoantibody re-evaluation is suggested (13, 14). We show that postpartum autoantibody re-measurement seems unwarranted in GDM women with borderline increased autoantibodies during pregnancy as these autoantibodies were negative or again only borderline increased at follow-up. In contrast, in GDM women with clinically significant increased autoantibodies during pregnancy, postpartum re-evaluation seems useful since autoantibodies further increased at follow-up. One woman, with clinical characteristics of the metabolic syndrome and no other autoimmune disorders, probably had false positive GADA during pregnancy (79.5 U/mL) as she developed glucose intolerance postpartum but without increased autoantibodies.

Overall, our data suggest that systematic screening for T1D-related autoantibodies in GDM women does not seem warranted since the low positivity rate for autoantibodies in pregnancy and postpartum. So far, there are no clear recommendations in which women with GDM it would be clinically relevant to screen for autoantibodies (13, 14). In our cohort, clinical and biochemical characteristics including insulin sensitivity and beta-cell function in pregnancy and postpartum were similar between GDM women with and without autoantibodies. We could therefore not uncover specific clinical and biochemical risk factors suggestive for autoimmune GDM. This is in line with most other studies, reporting no important dissimilarities in characteristics between both groups (14, 19, 45, 46). Only few studies could detect some characteristics associated with autoimmunity in GDM like younger age, lower BMI, lower fasting insulin level, and more frequent need for insulin therapy in pregnancy (16, 22, 47–49). These studies had several limitations, as in one study, all women with gestational hyperglycemia were considered, including women without GDM (16), while in two other studies, autoantibodies were measured at delivery (48, 49). In contrast with previous studies, we observed that GDM women with autoantibodies had more often gestational hypertension and more often neonatal hypoglycemia. This might suggest that besides hyperglycemia, autoimmunity might also affect the risk for adverse pregnancy outcomes. However, caution is warranted for interpretation of these study results due to the small sample size.

Previous studies have shown that the first two years postpartum seem to be the most critical for development of T1D (21, 49). This is in contrast with our findings, showing a very low risk for progression to T1D within 4 years postpartum (only one T1D diagnosis). We observed no differences in early postpartum rate of glucose intolerance between GDM women with and without autoantibodies. Moreover, we demonstrate that at long-term follow-up, presence of autoantibodies remained limited and only two women with glucose intolerance still showed autoantibody positivity while the majority with glucose intolerance was no longer autoantibody positive. However, rates can change rapidly and therefore an additional follow-up visit around 8-10 years postpartum might be recommended, especially in women with clinically significant increased autoantibodies in pregnancy and early postpartum.

The highest accuracy in predicting autoimmune diabetes seems to be achieved by screening with GADA (63% sensitivity) compared to ICA (48%) and IA-2A (34%), but single GADA appeared to have limited predictive power (21, 49). In our study, none showed GADA positivity at follow-up. Three women were positive for IA-2A (one with prediabetes at early postpartum and one with T1D). This confirms that IA-2A is associated with rapid β-cell dysfunction, indicating a higher risk of developing clinical signs within a shorter term (49). According to some studies, an increasing number of positive autoantibodies is highly predictive for progression towards T1D (13, 49). However, in our cohort, the only woman positive for two autoantibodies was still NGT at follow-up. In line with other studies, ZnT8A did not show additional predictive power for postpartum autoimmune diabetes in our study (13, 14, 17, 50).

A strength of this study is the availability of a large prospective cohort in pregnancy with long-term follow-up data, which allowed to evaluate various characteristics and biomarkers over time. Long-term follow-up data were available for most women with a history of GDM and autoantibodies in pregnancy from our cohort. Nevertheless, this involves a small group of GDM women with autoantibodies, though consistent with previous studies (14, 18). In future studies, estimation of optimal power for reliable statistical comparison between the groups is recommended. Another limitation is the lack of autoantibody re-evaluation in all women complicated with GDM at the early postpartum OGTT. Moreover, ZnT8A positivity was evaluated at follow-up but not during pregnancy as evidence about an association with T1D was limited at start of the study (51). Autoantibodies were not measured in women without GDM. However, in an Italian population, no significant difference in autoantibody positivity was found between women with and without GDM (5.6% vs. 8.3%, p=0.47) (14). In addition, no data were available on stimulated C-peptide. An association between autoimmunity against β-cells and other autoimmune disorders could not be assessed in our cohort due to limited data on other autoimmune diseases. Furthermore, our population was mainly Caucasian, indicating that our results might not be applicable to different ethnic populations.

## Conclusion

Systematic screening for T1D-related autoantibodies in GDM does not seem warranted since the low positivity rate for autoantibodies in pregnancy and postpartum. At 4.6 years postpartum, five out of 12 women were glucose intolerant but only two still had autoantibodies. The clinical characteristics, including insulin resistance and β-cell function were similar, independent of presence of autoantibodies. However, in women with clinically significant increased autoantibody levels during pregnancy, postpartum autoantibody re-measurement seems useful since the high risk for further increase of autoantibody levels.

## Data availability statement

The original contributions presented in the study are included in the article/**Supplementary Material**. Further inquiries can be directed to the corresponding author.

## Ethics statement

The studies involving human participants were reviewed and approved by Ethics Committee Research UZ/KU Leuven (EC Research). The patients/participants provided their written informed consent to participate in this study. Written informed consent was obtained from the individual(s) for the publication of any potentially identifiable images or data included in this article.

## Author contributions

KBen, PVC and CMa conceived the project. KBeu and CMo prepared the data and ALa did the statistical analysis. KBeu did the literature review. KBeu and KBen wrote the first draft of the manuscript. All authors contributed to the study design, including data collection, data interpretation and manuscript revision, and gave final approval to submit for publication.

## Funding

This investigator-initiated study was funded by the Belgian National Lottery, the Fund of the Academic studies of UZ Leuven, and the Fund Yvonne and Jacques François-de Meurs of the King Baudouin Foundation.

## Acknowledgments

KBen and RD are the recipient of a ‘Fundamenteel Klinisch Navorserschap FWO Vlaanderen’.

## Conflict of interest

The authors declare that the research was conducted in the absence of any commercial or financial relationships that could be construed as a potential conflict of interest.

## Publisher’s note

All claims expressed in this article are solely those of the authors and do not necessarily represent those of their affiliated organizations, or those of the publisher, the editors and the reviewers. Any product that may be evaluated in this article, or claim that may be made by its manufacturer, is not guaranteed or endorsed by the publisher.
