# Supplementary material for: Type 1 diabetes-related autoimmune antibodies in women with gestational diabetes mellitus and the long-term risk for glucose intolerance
Source: Front Endocrinol (Lausanne). 2022 Aug 24;13:973820. doi: 10.3389/fendo.2022.973820 (PMC9449803; doi:10.3389/fendo.2022.973820)
Supplement: Supplementary file 1 [file DataSheet_1.pdf]

Appendix 1. Comparison of characteristics and pregnancy outcomes between GDM with autoantibodies (group 1), GDM without autoantibodies (group 2) and NGT women (group 3).

	**GDM with autoantibodies N=15 (0.8%)**	**GDM without autoantibodies N=171 (9.5%)**	**NGT N=1612 (89.7%)**	**p-value 1 vs 2**	**1 vs 3**	**2 vs 3**
**General**						
Age (years)	32.9 ± 4.8	31.7 ± 4.4	30.6 ± 3.9	0.298	**0.024**	**<0.001**
% Minority ethnic background	13.3 (2)	16.5 (28)	8.2 (132)	1.000	0.357	**0.001**
% Multiparity	40.0 (6)	53.2 (91)	46.4 (748)	0.421	0.796	0.091
% Highest education:				0.792	0.910	0.315
Primary school	0.0 (0)	1.8 (3)	0.9 (15)			
Till 15 years	0.0 (0)	4.9 (8)	4.3 (69)			
High school	14.3 (2)	22.1 (36)	16.9 (269)			
Bachelor	42.9 (6)	36.2 (59)	42.0 (667)			
Master	42.9 (6)	35.0 (57)	35.8 (568)			
% Paid job	100.0 (15)	89.9 (151)	91.8 (1474)	0.367	0.627	0.381
% Low monthly net income family				0.575	0.740	0.307
<1500 euro	0.0 (0)	6.0 (10)	4.1 (64)			
1500-5000 euro	92.9 (13)	89.8 (149)	89.7 (1415)			
>5000 euro	7.1 (1)	4.2 (7)	6.2 (98)			
% Smoking before pregnancy	26.7 (4)	40.8 (69)	28.5 (457)	0.410	1.000	**0.001**
% Smoking during pregnancy	0.0 (0)	6.5 (11)	3.2 (52)	0.604	1.000	**0.045**
% First degree family history of diabetes	6.7 (1)	19.8 (33)	11.8 (185)	0.310	1.000	**0.005**
% First degree family history of GDM	0.0 (0)	8.2 (13)	4.0 (60)	0.602	1.000	**0.022**
% History of GDM [c]	16.7 (1)	30.0 (27)	5.3 (40)	0.668	0.282	**<0.001**
% History of IGT [a]	0.0 (0)	3.3 (5)	1.1 (15)	1.000	1.000	**0.043**
% History of macrosomia >4 kg	6.7 (1)	8.2 (14)	5.8 (93)	0.716	0.757	0.150
**6-14 weeks visit**						
Gestational age first visit	11.8 ± 1.5	11.9 ± 1.6	11.9 ± 1.8	0.893	0.861	0.870
BMI (kg/m²)	27.0 ± 5.4	26.7 ± 5.4	24.4 ± 4.5	0.868	**0.027**	**<0.001**
% Overweight	26.7 (4)	31.2 (53)	24.8 (398)	0.970	0.114	**<0.001**
% Obesity	26.7 (4)	24.1 (41)	11.0 (177)			
Waist circumference (cm)	90.1 ± 12.4	91.5 ± 12.8	86.5 ± 10.9	0.683	0.204	**<0.001**
% Waist ≥80 cm	93.3 (14)	81.5 (132)	74.1 (1144)	0.156	0.196	**0.002**
Weight gain (first visit-OGTT) (kg)	7.4 ± 2.7	6.7 ± 3.3	7.1 ± 3.3	0.467	0.775	0.136
Systolic blood pressure (mmHg)	117.4 ± 9.1	116.8 ± 11.8	114.8 ± 10.4	0.841	0.335	**0.020**
Diastolic blood pressure (mmHg)	73.5 ± 7.0	72.1 ± 8.7	70.3 ± 8.1	0.558	0.134	**0.001**
Total Score lifestyle						
Physical activity	0.0 (0.0 – 2.0)	1.0 (0.0 – 2.0)	1.0 (0.0 – 2.0)	0.909	0.663	0.391
Diet	2.0 (0.0 – 3.0)	2.0 (0.0 – 4.0)	2.0 (0.0 – 4.0)	0.590	0.552	0.975
Fasting glycemia (mg/dL)	86.0 (84.0 – 90.0)	85 (80.0 – 89.0)	81 (78.0 - 85.0)	0.242	**<0.001**	**<0.001**
HOMA-IR	10.0 (9.5 – 19.9)	10.9 (7.9 – 16.8)	9.1 (6.5 – 12.9)	0.689	**0.040**	**<0.001**
HOMA-B	888.6 (641.7 – 1486.3)	927.7(662.4 – 1318.5)	918.0 (667.8 – 1284.0)	0.946	0.910	0.575
HbA1c (mmol/mol and %)	32.0 (30.0 – 36.0) 5.1 (4.9 - 5.4)	32.0 (30.0 – 34.0) 5.1 (4.9 – 5.3)	31.0 (29.0 – 32.0) 5.0 (4.8 – 5.1)	0.495	**0.044**	**<0.001**
Fasting total cholesterol (mg/dL)	177.0 (157.0 – 205.0)	186.0 (164.0 – 211.0)	180.0 (161.0 – 203.0)	0.252	0.606	**0.017**
Fasting HDL (mg/dL)	73.0 (55.0 – 79.0)	67.0 (59.0 – 76.0)	68.0 (60.0 – 77.0)	0.575	0.740	0.468
Fasting LDL (mg/dL)	86.0 (69.0 – 111.0)	96.0 (82.0 – 115.0)	93.0 (77.0 – 111.0)	0.130	0.318	0.046
Fasting TG (mg/dL)	104.0 (70.0 – 118.0)	98.0 (81.0 – 133.0)	88.0 (71.0 – 111.0)	0.603	0.280	**<0.001**
**24-28 weeks visit**						
BMI (kg/m²)	29.0 ± 5.1	29.3 ± 5.3	26.9 ± 4.4	0.854	0.084	**<0.001**
% Overweight	42.9 (6)	42.1 (69)	40.1 (629)	0.855	0.677	**<0.001**
% Obesity	28.6 (4)	37.2 (61)	21.1 (332)			
Systolic blood pressure (mmHg)	116.7 ± 10.3	115.4 ± 11.7	113.1 ± 10.1	0.670	0.167	**0.006**
Diastolic blood pressure (mmHg)	72.0 ± 8.1	69.1 ± 8.1	67.0 ± 7.9	0.189	**0.015**	**<0.001**
Total Score lifestyle						
Physical activity	1.0 (0.0 – 2.0)	1.0 (0.0 – 2.0)	1.0 (0.0 – 2.0)	0.487	0.538	0.942
Diet	1.0 (0.0 – 5.0)	2.0 (-1.0 – 4.0)	2.0 (0.0 – 4.0)	0.684	0.888	0.228

	GDM with autoantibodies N=15 (0.8%)	GDM without autoantibodies N=171 (9.5%)	NGT N=1612 (89.7%)	p-value 1 vs 2	1 vs 3	2 vs 3
METs categories:				**0.031**	**0.035**	0.590
% Low	0.0 (0)	19.4 (32)	16.3 (252)			
% Moderate	33.3 (5)	45.4 (75)	47.2 (731)			
% High	66.7 (10)	35.1 (58)	36.5 (566)			
Gestational age GCT	24.8 ± 1.3	24.5 ± 1.1	24.5 ± 0.9	0.367	0.236	0.880
Glucose non-fasting 0 min on GCT (mg/dL)	105.0 ± 27.4	98.0 ± 20.5	88.0 ± 15.8	0.218	**0.004**	**<0.001**
Glucose 60 min on GCT (mg/dL)	145.9 ± 21.5	143.4 ± 29.7	116.9 ± 25.5	0.754	**<0.001**	**<0.001**
Fasting glycemia (mg/dL)	91.0 (84.0 – 94.0)	85.0 (78.0 – 92.0)	78.0 (74.0 – 82.0)	0.081	**<0.001**	**<0.001**
30 min glucose OGTT (mg/dL)	160.0 (130.0 – 177.0)	149.0 (134.0 – 163.0)	124.0 (112.0 – 137.0)	0.381	**<0.001**	**<0.001**
1-hour glucose OGTT (mg/dL)	178.0 (157.0 – 192.0)	172.5 (154.0 – 186.0)	123.0 (107.0 – 141.0)	0.241	**<0.001**	**<0.001**
2-hour glucose OGTT (mg/dL)	151.0 (142.0 – 169.0)	156.0 (134.0 – 167.0)	108.0 (92.0 – 124.0)	0.850	**<0.001**	**<0.001**
HbA1c (mmol/mol and %)	32.0 (30.0 – 34.0) 5.1 (4.9 – 5.3)	32.0 (30.0–34.0) 5.1 (4.9 – 5.3)	30.0 (29.0 – 32.0) 4.9 (4.8 – 5.1)	0.786	**0.005**	**<0.001**
Matsuda insulin sensitivity	0.3 (0.3 – 0.4)	0.4 (0.2 – 0.5)	0.6 (0.4 – 0.8)	0.510	**<0.001**	**<0.001**
HOMA-IR	17.0 (13.4 – 21.4)	17.3 (11.3 – 28.5)	11.9 (8.6 – 16.8)	0.986	**0.003**	**<0.001**
HOMA-B	1184.0 (814.1 – 1617.4)	1433.8 (1046.4 – 2065.1)	1591.3 (1133.9 – 2286.7)	0.115	**0.016**	**0.013**
ISSI-2	0.08 (0.05 – 0.11)	0.09 (0.04 – 0.15)	0.14 (0.08 – 0.25)	0.587	**0.003**	**<0.001**
Insulinogenic index/HOMA-IR	0.2 (0.1 – 0.3)	0.2 (0.2 – 0.3)	0.3 (0.2 – 0.5)	0.682	**<0.001**	**<0.001**
Fasting total cholesterol (mg/dL)	243.0 (224.0 – 259.0)	245.0 (218.0 – 273.0)	243.0 (219.0 – 273.0)	0.575	0.705	0.504
Fasting HDL (mg/dL)	74.0 (71.0 – 88.0)	73.0 (62.0 – 84.0)	74.0 (64.0 – 86.0)	0.113	0.310	0.074
Fasting LDL (mg/dL)	124.0 (109.0 – 141.0)	135.0 (109.0 – 156.0)	133.0 (112.0 – 161.0)	0.280	0.256	0.999
Fasting TG (mg/dL)	179.0 (145.0 – 203.0)	184.0 (147.0 – 233.0)	160.0 (128.0 – 202.0)	0.513	0.241	**<0.001**
% Need for treatment with insulin (total)	6.7 (1)	15.8 (27)	NA	0.623	NA	NA
% Short acting insulin	0.0 (0)	6.4 (11)				
% Long acting insulin	0.0 (0)	4.1 (7)				
% Short and long-acting insulin	6.7 (1)	5.3 (9)				
**Delivery**						
Total weight gain (first visit-delivery) (kg)	8.3 ± 4.6	8.6 ± 5.0	12.2 ± 5.0	0.803	**0.003**	**<0.001**
% Excessive weight gain	26.7 (4)	18.4 (28)	30.9 (437)	0.573	0.142	**<0.001**
Gestational age (weeks)	38.4 ± 1.6	38.9 ± 1.5	39.3 ± 1.6	0.281	**0.040**	**0.001**
% Preeclampsia	6.7 (1)	1.2 (2)	1.8 (29)	0.224	0.245	0.762
% Gestational hypertension	33.3 (5)	1.7 (3)	4.2 (68)	**<0.001**	**<0.001**	0.148
% Preterm delivery	20.0 (3)	6.4 (11)	5.4 (86)	0.090	**0.045**	0.594
% Induction labor	46.7 (7)	36.3 (62)	25.9 (416)	0.420	0.079	**0.005**
% Forceps or vacuum	13.3 (2)	11.1 (19)	12.3 (198)	0.679	0.707	0.713
% CS (total)	26.7 (4)	29.2 (50)	20.2 (324)	1.000	0.522	**0.008**
% Planned CS	13.3 (2)	12.9 (22)	10.2 (164)	1.000	0.661	0.293
% Emergency CS (during labor)	13.3 (2)	16.4 (28)	10.0 (160)	1.000	0.656	**0.013**
Weight baby (g)	3217.7 ± 620.0	3337.4 ± 471.9	3397.9 ± 509.9	0.360	0.174	0.138
% Macrosomia (>4 kg)	0.0 (0)	8.2 (14)	151 (9.4)	0.609	0.387	0.679
% Weight baby ≥4.5 kg	0.0 (0)	0.0 (0)	1.3 (21)		1.000	0.254
% LGA	6.7 (1)	14.6 (25)	12.9 (206)	0.698	0.709	0.550
% SGA	6.7 (1)	5.3 (9)	5.1 (81)	0.578	0.544	0.855
% Apgar 10 min <7	0.0 (0)	0.6 (1)	0.9 (15)	1.000	1.000	1.000
% Shoulder dystocia	0.0 (0)	1.2 (2)	1.1 (18)	1.000	1.000	1.000
% Congenital anomaly	6.7 (1)	5.9 (10)	4.3 (68)	1.000	0.484	0.325
% Respiratory distress syndrome	0.0 (0)	1.2 (2)	0.9 (15)	1.000	1.000	0.677
% Neonatal hypoglycemia <40 mg/dL [b]	40.0 (6)	12.5 (19)	4.0 (41)	**0.012**	**<0.001**	**<0.001**
Neonatal jaundice [b]	14.3 (2)	15.7 (17)	18.9 (216)	1.000	1.000	0.518
% NICU admission	20.0 (3)	15.2 (26)	9.6 (153)	0.708	0.171	**0.032**

GDM gestational diabetes mellitus; NGT normal glucose tolerance; IGT impaired glucose tolerance; BMI Body Mass Index; OGTT oral glucose tolerance test; HOMA-IR Homeostatic Model Assessment for Insulin Resistance; HOMA-B Homeostatic Model Assessment for β-cell function; HbA1c glycated hemoglobin; HDL high-density lipoprotein cholesterol; LDL low-density lipoprotein cholesterol; TG triglycerides; MET metabolic equivalent of task; GCT glucose challenge test; ISSI-2 insulin secretion-sensitivity index-2; CS caesarean section; LGA large for gestational age infant; SGA small for gestational age infant; NICU neonatal intensive care unit. Overweight BMI ≥25-29.9 kg/m^2; Obesity BMI ≥30 kg/m^2. Categorical variables are presented as frequencies % (n); continuous variables are presented as mean ± SD if normally distributed and as median ± IQR if not normally distributed; differences are considered significant at p-value <0.05 and are indicated in bold.

[a] For these variables, data were missing in 10–15% of all participants.

[b] For these variables, data were missing in 25–35% of all participants.

[c] For these variables, data were missing in 50–55% of all participants.

Appendix 2. Comparison of additional characteristics between GDM with autoantibodies and GDM without autoantibodies at the early postpartum OGTT.

	**GDM with autoantibodies N=14 (93.3%)**	**GDM without autoantibodies N=153 (89.5%)**	**p-value**
Fasting insulin (pmol/L)	46.1 (42.4 – 84.2)	52.6 (36.2 – 83.7)	0.911
30 min insulin OGTT (pmol/L)	370.8 (293.6 – 648.0)	377.8 (258.2 – 502.3)	0.625
1-hour insulin OGTT (pmol/L)	508.7 (346.8 – 925.3)	421.9 (286.3 – 635.4)	0.319
2-hour insulin OGTT (pmol/L)	333.5 (256.5 – 397.5)	312.0 (221.1 – 497.6)	0.942
1/HOMA-IR	0.1 (0.0 – 0.1)	0.1 (0.06 – 0.1)	0.861
Lifestyle score:			
Physical activity	1.0 (0.0 – 3.0)	1.0 (0.0 – 2.0)	0.803
Diet	2.0 (0.0 – 3.0)	2.0 (0.0 – 4.0)	0.848
SF-36			
Physical functioning	95.0 (80.0 – 100.0)	90.0 (75.0 – 100.0)	0.501
Role physical	87.5 (75.0 – 100.0)	87.5 (68.7 – 100.0)	1.000
Role Emotional	100.0 (50.0 – 100.0)	100.0 (66.7 – 100.0)	0.715
Energy	65.6 (56.2 – 75.0)	62.5 (50.0 – 75.0)	0.898
Emotional Wellbeing	70.0 (62.5 – 75.0)	70.0 (65.0 – 80.0)	0.532
Social functioning	87.5 (75.0 – 100.0)	87.5 (75.0 – 100.0)	0.944
Pain	100.0 (80.0 – 100.0)	90.0 (77.5 – 100.0)	0.411
General Health	80.0 (50.0 – 85.0)	75.0 (65.0 – 85.0)	0.472
Health Transition	50.0 (50.0 – 50.0)	50.0 (50.0 – 50.0)	0.672
METs category:			0.365
% Low	21.4 (3)	9.3 (13)	
% Moderate	42.9 (6)	50.0 (70)	
% High	35.7 (5)	40.7 (57)	
% Clinical depression (≥16 on CES-D questionnaire)	26.7 (4)	15.9 (24)	0.286

GDM gestational diabetes mellitus; OGTT oral glucose tolerance test; HOMA-IR Homeostatic Model Assessment for Insulin Resistance; SF-36 36-Item Short Form Health Survey; MET metabolic equivalent of task; CES-D Center for Epidemiologic Studies – Depression. Questionnaires in the early postpartum period were only administered by women with GDM who attended the OGTT. Categorical variables are presented as frequencies % (n); continuous variables are presented as mean ± SD if normally distributed and as median ± IQR if not normally distributed; differences are considered significant at p-value <0.05 and are indicated in bold.

Appendix 3. Characteristics of GDM women with autoantibodies at the early postpartum and long-term follow-up OGTT.

	**1**	**2**	**3**	**4**	**5**	**6**	**7**	**8**	**9**	**10**	**11**	**12**
**At the early postpartum OGTT**												
Weight (kg)	61.4	78.6	53.2	66.7	63.1	62.0	83.3	89.6	75.9	108.1	88.6	57.2
BMI (kg/m²)	21.5	25.4	21.0	25.1	30.9	24.2	33.4	31.4	25.7	37.4	28.9	22.3
Waist circumference (cm)	75.0	92.0	/	81.0	87.0	87.0	105.0	108.0	89.0	120.0	106.0	81.0
Systolic blood pressure (mmHg)	122	111	112.5	117.5	102	109.5	121.5	117.5	114.5	119	111.5	121.5
Diastolic blood pressure (mmHg)	75	61	70.5	68	73.5	66	83	76	74.5	88	65.5	76
Fasting glycaemia (mg/dL)	84	86	78	87	108	87	92	91	91	86	86	108
30 min glucose OGTT (mg/dL)	186	106	138	137	177	156	121	130	155	135	163	123
1-hour glucose OGTT (mg/dL)	111	91	157	115	157	156	146	134	182	131	165	154
2-hour glucose OGTT (mg/dL)	96	68	102	88	105	111	138	121	109	68	91	100
Glucose tolerance	NGT	NGT	NGT	NGT	IFG (pre-diabetes)	NGT	NGT	NGT	NGT	NGT	NGT	IFG (pre-diabetes)
HbA1c (mmol/mol and %)	30<br>4.9	34<br>5.3	32<br>5.1	36<br>5.4	37<br>5.5	37<br>5.5	41<br>5.9	32<br>5.1	40<br>5.8	31<br>5.0	33<br>5.2	31<br>5.0
**At the follow-up OGTT**												
Weight (kg)	63.1	75.6	/	78	57.6	70.9	99.6	88.8	86	115	107.6	67
BMI (kg/m²)	22.4	24.4	/	29.7	26.7	27.7	39.9	31.5	29.1	39.8	35.3	27.2
Waist circumference (cm)	78.8	78.8	/	83.7	80.5	82.5	106.8	107	/	122	/	/
Systolic blood pressure (mmHg)	132	110	/	147	138	137	149	118	130	145	/	/
Diastolic blood pressure (mmHg)	78	74	/	87	85	88	88	85	77	90	/	/
Fasting glycaemia (mg/dL)	93	92	81	95	101	98	110	95	90	83	101	94
30 min glucose OGTT (mg/dL)	129	167	125	163	110	176	155	131	166	113	179	141
1-hour glucose OGTT (mg/dL)	75	145	153	162	136	212	179	163	163	114	244	193
2-hour glucose OGTT (mg/dL)	117	97	109	113	101	200	150	135	142	107	204	197
Glucose tolerance	NGT	NGT	NGT	NGT	IFG (pre-diabetes)	IGT (T1D)	IFG+IGT (pre-diabetes)	NGT	NGT	NGT	IFG+IGT (T2D)	IGT (pre-diabetes)

HbA1c (mmol/mol and %)	34<br>5.3	38<br>5.6	36<br>5.4	40<br>5.8	37<br>5.5	36<br>5.4	43<br>6.1	31<br>5.0	38<br>5.6	33<br>5.2	/	/
Fasting C-peptide (nmol/L)	3.4	3.2	0.4	2.3	1.6	2.8	1.9	0.6	/	0.8	/	/
Matsuda insulin sensitivity	0.7	0.3	0.7	0.2	0.4	0.3	0.2	/	/	0.4	/	/
HOMA-IR	10.4	22.4	9.4	28.4	26.7	15.0	62.2	/	/	17.6	/	/
1/HOMA-IR	0.1	0.04	0.1	0.03	0.04	0.1	0.02	/	/	0.06	/	/
HOMA-B	546	1226.5	936.0	1361.2	1013.7	636.7	1754.0	/	/	1547.82	/	/
Insulinogenic index/HOMA-IR	0.6	0.2	0.2	0.2	0.1	0.2	0.1	/	/	0.3	/	/
ISSI-2	0.2	0.05	0.2	0.04	0.1	0.05	0.03	/	/	0.1	/	/
Stumvoll index	-82.8	1306.6	1.2	1209.2	668.5	752.3	1129.6	/	/	786.0	/	/

OGTT oral glucose tolerance test; BMI Body Mass Index; NGT normal glucose tolerance; IFG impaired fasting glucose; HbA1c glycated hemoglobin; IGT impaired glucose tolerance; T1D type 1 diabetes mellitus; T2D type 2 diabetes mellitus; HOMA-IR Homeostatic Model Assessment for Insulin Resistance; HOMA-B Homeostatic Model Assessment for β-cell function; ISSI-2 insulin secretion-sensitivity index-2. Numbers 1-12 represent the GDM women with autoantibodies that also had a long-term follow-up visit.
